# Effectiveness of Laser Therapy in Treatment of Temporomandibular Joint and Muscle Pain

**DOI:** 10.3390/jcm13175327

**Published:** 2024-09-09

**Authors:** Smaranda Buduru, Denisa Maria Oprea, Manuela-Maria Manziuc, Daniel-Corneliu Leucuța, Oana Almășan

**Affiliations:** 1 Prosthetic Dentistry and Dental Materials Department, Iuliu Hațieganu University of Medicine and Pharmacy, 32 Clinicilor Street, 400006 Cluj-Napoca, Romania; 2 Department of Medical Informatics and Biostatistics, Iuliu Hațieganu University of Medicine and Pharmacy, 400349 Cluj-Napoca, Romania

**Keywords:** temporomandibular disorders, masticatory muscle pain, laser treatment

## Abstract

**Background/Objectives:** Temporomandibular joint disorders (TMDs) express a condition derived from a broad spectrum of etiological factors and clinical manifestations. Many treatment options have been developed for TMDs; nevertheless, conservative and non-invasive approaches ought to be prioritized. Laser therapy is an effective treatment for pain management due to its non-invasive nature and capacity for tissue regeneration. This review aimed at bringing an overview of the present evidence regarding the efficiency of laser therapy on myofascial or temporomandibular joint disorders pain. **Methods:** The search was conducted in four electronic databases: PubMed, Web of Science, Embase, and Scopus, of studies published between January 1997 and January 2023. The following terms have been extensively searched: “laser treatment”, pain management”, “temporomandibular joint disorders”, “masseter muscle pain”, “pterygoid muscle pain”, and “temporal muscle pain”. The inclusion criteria were original papers, available in full text, and written in English. Cohen’s Kappa coefficient was used to assess the inter-rater reliability for article selection. The methodological quality was assessed with the Cochrane Risk of Bias tool for randomized controlled trials and the National Heart, Lung, and Blood Institute’s quality assessment tool for before-after studies with no control group. **Results:** Out of 846 identified records, 7 studies were included, of which 5 were randomized controlled trials. The inter-rater reliability for article selection showed an almost perfect agreement (Cohen’s Kappa = 0.832, *p* < 0.001). The protocol of laser application was not standardized; the laser wavelength ranged from 633 to 940 nm, with a power output range from 25 to 1600 mW. The number of sessions varied from 3 to 12, with a frequency of application from 1 time per week to 3 times per week. All studies reported pain reduction after laser therapy. **Conclusions:** Laser therapy is an efficient method to treat TMDs related to muscle pain. To accomplish the desired results, a standard procedure must be followed; however, the protocol is still not fully designed.

## 1. Introduction

Temporomandibular joint disorders (TMDs) are conditions that affect the temporomandibular joint (TMJ), resulting in pain, disc displacement, and overall discomfort and dysfunction of the surrounding muscles [1]. Symptoms and signs of this pathology include clicking or popping sounds, discomfort or pain during jaw opening or closing, limitation of jaw opening, difficulty in mastication, otalgia, and headaches [2]. TMDs can be caused by a variety of etiological factors, including teeth grinding, bruxism, and psychological stress [3,4]. One of the main characteristics of TMD is represented by pain, which may be acute or chronic, representing the most important symptom and primary reason for patients to consult a clinician. Over the past years, the diagnostic criteria for TMD have been significantly developed to provide the most accurate diagnosis and treatment for the patients. According to the Diagnostic Criteria for Temporomandibular Disorder (DC/TMD), this medical condition is classified into different categories, such as muscle disorders, internal derangements of the joint, arthralgia, and degenerative joint diseases [5], which may have a very complex etiology such as genetic, hormonal, or psychological [6,7,8]. TMDs can occur at any age, yet the most common is in early adulthood. Most patients seeking specialized treatment are women, outnumbering men by 4 to 1. Muscle disorders are defined by pain in the masticatory muscles during different functions (chewing, speaking, laughing), or clinical palpation, or even at rest, but may also involve limited mandibular movements or decreased mouth opening amplitude. Among all the masticatory muscles, most frequently, the masseter and temporalis muscles are associated with TMDs. At present, there is a large variety of treatment options, but the treatment of choice should be conservative and non-invasive to re-establish the muscle function and relieve pain [9].

Low-level laser therapy (LLLT) is used for various conditions such as arthritis, wound healing, or musculoskeletal pain, and is also highly recommended for the treatment of TMD. LLLT has significant therapeutic results, determined by its unique properties such as biostimulation effect, tissue regeneration, anti-inflammatory effect, and pain relief [10]. 

LLLT employs different wave intensities to stimulate cellular function (low-, medium-, high-intensity lasers) [10]. Low-level laser therapy exerts its mechanism through a photochemical effect compared to medical laser procedures, which are associated with a thermal or ablative effect on tissues. LLLT has an analgesic effect that can be explained by the following: an increase in ATP production, a decrease in bradykinin and histamine release, increased blood supply, edema reduction, muscle relaxation, and an increase in beta-endorphin and serotonin levels [11]. Numerous benefits are achieved, such as release of endogenous opioids, tissue repair, a decrease in inflammation, and an increase in vasodilation and pain threshold [12]. In some cases, a combination of different laser wavelengths can increase their positive outcomes compared to individual wavelengths [13,14,15].

Due to the complexity of TMDs, the diagnosis and treatment plan may sometimes be challenging for clinicians. Considering the multifactorial etiology of TMDs, a universal and ideal treatment option has not been established. Consequently, different conventional and innovative treatment options can be used to achieve good long-term results [16,17].

This study aimed at systematically reviewing the current research regarding the influence of laser therapy on temporomandibular disorders.

## 2. Materials and Methods

The research approach was prepared in accordance with the Preferred Reporting Items for Systematic Reviews and Meta-Analyses (PRISMA). The databases Scopus, Embase, PubMed, and Web of Science were reviewed from January 1997 to January 2023. The following keywords were used: “laser treatment”, “pain management”, “temporomandibular joint disorders”, “masseter muscle pain”, “pterygoid muscle pain”, and “temporal muscle pain”. For the search strategies, the search terms were introduced in all the fields as well as the MeSH terms for PubMed and Emtree terms for Embase. Also, the singular and plural forms, synonyms, and abbreviations were used to construct the strategies. The inclusion criteria were original papers available in full text and written in English. The exclusion criteria were animal studies, case reports, case series, systematic reviews, narrative reviews, and studies that did not correlate with the purpose of this review. The inter-rater reliability for article selection was assessed with Cohen’s Kappa coefficient (Jamovi version 2.3.28). The search strategy is shown in Table 1. The included studies were assessed for their methodological quality with the Cochrane Risk of Bias tool for randomized controlled trials and the National Heart, Lung, and Blood Institute’s quality assessment tool for before-after studies with no control group. 

## 3. Results

### Study Selection

**Figure 1:**
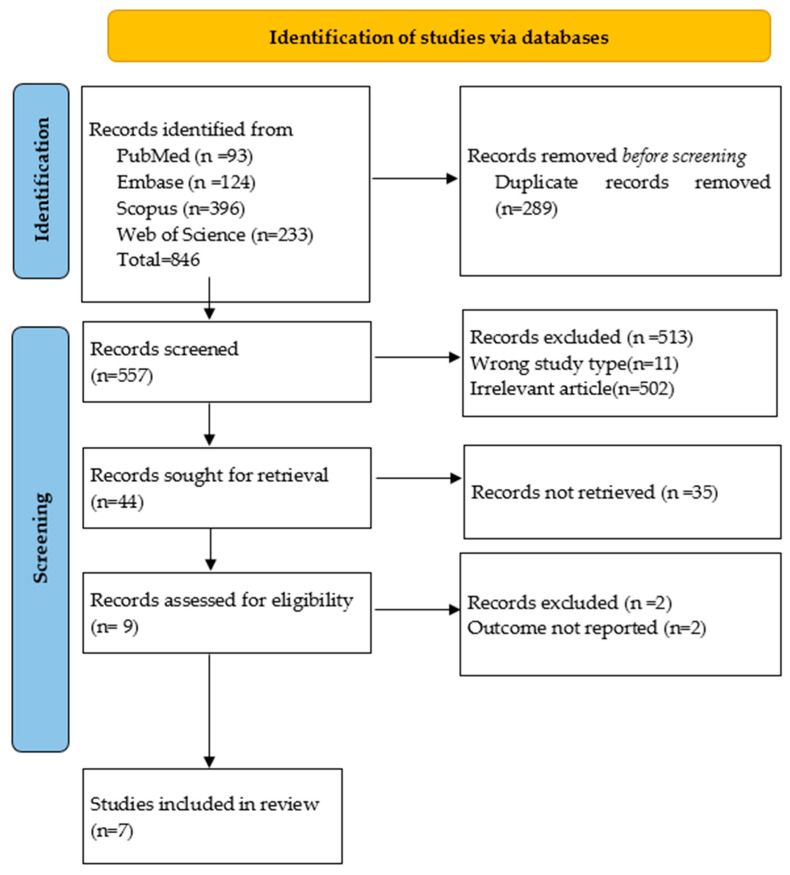
**The** PRISMA Flowchart of the identification, screening, and inclusion of the articles for the review.

The MEDLINE and EMBASE searches for temporomandibular joint disorders, myofascial pain, and laser therapy provided a total number of 846 articles. Two reviewers (D.M.O., M.-M.M.) independently assessed the selection criteria for each chosen article. Inter-rater reliability for article selection was assessed, and Cohen’s Kappa was 0.832, *p* < 0.001, showing an almost perfect agreement. After the exclusion of duplicates, the total number of potentially relevant articles was 557. In cases of disagreement, consensus was established with a third reviewer (D.-C.L.). Next, the titles and abstracts of each article were analyzed to determine if they were admissible for the study; as a result, 513 were excluded. After this process, 44 studies were potentially relevant. In total, 7 articles were included in the study, with a number of 324 participants. A PRISMA flow diagram chart was used to illustrate the selection process (Figure 1).

## 4. Study Characteristics

Study characteristics are presented in Table 2 and Table 3. Table 2 shows the characteristics of each type of laser used by clinicians for the treatment of TMD or myofascial pain in the studies included in this research. 

A range of laser types and wavelengths is used in the investigations on laser treatment for myofascial pain and TMD, which illustrates the diversity of methods associated with this area of study. Al-Quisi et al. [18] combined two forms of light therapy by using dual wavelengths: 660 nm for red LED light and 810 nm for LASER. An 820 nm low-intensity laser was employed by Sancakli et al. [19] and a 795 nm infrared laser was used by Uemoto et al. [20]. Huang et al. [21] used an 800 nm laser; however, they did not specify the type. By combining GaAlAs lasers with SLD red light at 633 nm and 785 nm in wavelength, Nencheva-Svechtarova et al. [22] provided a dual-wavelength method. A 905 nm gallium arsenide laser was employed by Nambi et al. [24], and a 940 nm low-level gallium arsenide diode laser was used by Shousha et al. [23]. The diversity of laser varieties and wavelengths highlights the unpredictability of the state of the art and the continuous effort to maximize the benefits of therapeutic approaches using lasers.

Research findings show substantial differences in the power outputs of lasers used for treating myofascial pain and TMDs. Al-Quisi et al. [18] recorded the maximum power outputs, using 1000 mW for a laser and 1600 mW for LED lights. Huang et al. [21] employed a 1500 mW power output, and Sancakli et al. [19] a 300 mW laser. Despite having various specs and wavelengths, Nencheva-Svechtarova et al. [22] and Shousha et al. [23] used 200 mW power output lasers. A laser with an output of 80 mW was employed by Uemoto et al. [20]. Nambi et al. used the lowest power output of 25 mW [24] and employed a gallium arsenide super pulsed laser.

The different methodologies and therapeutic procedures in current research are reflected in a broad spectrum of power outputs, underscoring the necessity for additional investigation to identify the optimal intensity levels for laser therapy in the management of TMD and myofascial pain.

The length of time and frequency of laser treatments for myofascial pain and TMD vary greatly throughout research. In 3 sessions spread over 4 weeks, Al-Quisi et al. [18] employed 300 s for LED and 30 s for LASER for each location. Throughout 12 sessions, consisting of 3 sessions each week for 4 weeks, Sancakli et al. [19] used 10 s for each point. While Uemoto et al. [20] performed 4 sessions over 2 weeks, they did not quantify the length of each session. In their 2014 study, Huang et al. [21] employed weekly sessions until recuperation, averaging 134 s at each point. Throughout 6 sessions spread across 2 weeks, Nencheva-Svechtarova et al. [22] had 100 to 300 s for each point. Using 3 sessions every week, Shousha et al. [23] applied 10 s for each point across 10 sessions. Nambi et al. [24] employed two techniques in 12 sessions spread over 4 weeks: 240 s transversely and 60 s in four locations. This diversity suggests different strategies to maximize the effectiveness of therapy.

Table 3 offers brief information about the main characteristics of each study.

The various studies on laser therapy for TMDs and myofascial pain employed diverse methodologies and criteria, but collectively they illustrate the treatment’s potential benefits. Subjects with TMD or myofascial pain were typically selected as part of the inclusion criteria, incorporating symptoms like acute myofascial trigger points. Recent trauma, ongoing medical interventions, systemic illnesses, and psychological disorders were frequently cited as exclusion factors. The Visual Analog Scale (VAS) was a common tool used in evaluation procedures; however, several studies also included other measurements, such as surface EMG, mandibular movement assessments, and pain pressure threshold evaluations. The results showed that there were no appreciable variations in the effects of various laser types and protocols, and that laser therapy consistently reduced pain and enhanced mandibular function when used in conjunction with other therapies such as lidocaine injections. These findings suggest that laser therapy is an effective treatment modality for reducing pain and improving function in patients with TMDs and myofascial pain.

The risk of bias assessment for five randomized controlled trials (RCTs) reveals diverse levels of methodological rigor (Table 4). For random sequence generation, most studies (Al Quisi et al. [18], Sancakli et al. [19], Shousha et al. [23], Nambi et al. [24]) show a low risk, except for Uemoto et al. [20], which has a high risk. Allocation concealment presents a high risk for most studies except Shousha et al. [23]. Performance bias, assessed through blinding of participants and personnel, is low in Al Quisi et al. [18], Sancakli et al. [19], and Shousha et al. [23], but high in Uemoto et al. [20] and Nambi et al. [24]. Detection bias for patient-reported outcomes is low in four studies but high in Uemoto et al. [20]; for objective outcomes, it is low in three studies, high in Uemoto et al. [20], and not applicable to Al Quisi et al. [18]. All studies have a low risk for attrition bias in short-term outcomes, while only Nambi et al. [24] assessed longer-term outcomes, reporting a low risk. Selective reporting shows a low risk in Al Quisi et al. [18], Shousha et al. [23], and Nambi et al. [24], but a high risk in Sancakli et al. [19] and Uemoto et al. [20]. Overall, while some studies demonstrate strong methodological rigor and low bias, others exhibit significant risks that could affect the reliability of their findings.

The quality of two before-after studies without control groups by Huang et al. [21] and Nencheva-Svechtarova et al. [22] was assessed by using the National Heart, Lung, and Blood Institute’s quality assessment tool (Table 5). Both studies clearly stated their objectives and described their eligibility criteria, had sufficiently large sample sizes, and consistently described and delivered the interventions. They also used prespecified, valid, and reliable outcome measures and appropriate statistical methods to assess pre-to-post intervention changes. However, it is unclear if the participants were representative of the general population or if all eligible participants were enrolled. Both studies did not blind outcome assessors, and Nencheva-Svechtarova et al. [22] had issues with follow-up accounting. 

## 5. Discussion

Orofacial pain may involve a wide spectrum of clinical conditions, including dental-induced orofacial pain and musculoskeletal orofacial pain, which may not be caused by a particular dental condition. Therefore, clinicians should carefully consider all the signs and symptoms described by patients to accurately determine whether the pain originates from the teeth or has a more complex cause [25].

Laser therapy represents a safe therapeutic option for the treatment of temporomandibular joint pain and myofascial pain. The World Association for Photobiomodulation Therapy (WALT) promotes research and clinical applications by adopting protocols that involve control groups to effectively measure the final clinical outcomes [10]. LLLT in the infrared spectrum (with a wavelength of about 700 nm) could penetrate 3 to 5 cm into the tissue and has an affinity for nerves, thus inducing a photochemical effect on irradiated cells and reducing the pain, compared to red lasers (with a wavelength between 600 and 700 nm), which are poorly absorbed into the tissue, therefore being less capable of targeting the nervous cells. Additionally, based on the power, it has been shown that low-power lasers (less than 250 mW) do not modify the tissue temperature; therefore, they act only through photobiomodulation [11].

Al-Quisi et al. [18] showed in their study that both LED light and LASER reduced the pain value and the number of trigger points (masseter, temporalis, and pre-auricular muscles), but no significant variations were found between the two treatment options.

Other researchers employed an 820 nm laser with a power output of 300 mW, administered for 10 s at each trigger point, dividing the patients into two different categories [19]. The first group of patients received continuous and precise laser treatment on fascicles of the masseter and/or temporalis muscles, while for the second group, the laser therapy was performed separately for each muscle fascicle, such as superior, middle, and inferior masseter and anterior, middle and posterior for temporalis. At the end of the therapy sessions, a significant reduction in pressure pain values was reported for both groups, together with a decreased pain intensity during muscle palpation and an improvement in mandibular movements [19].

Different values of the laser power output were assessed. Huang et al. [21] used an 800 nm laser with a power output of 1.5 W for 134 s per point once a week until patients recovered. They concluded that the patients felt facial pain relief after three treatment sessions. When comparing the therapeutic results of two different types of lasers, Nencheva-Svechtarova et al. [22] demonstrated that GaAlAs laser diodes (785 nm) and SLD red light (633 nm) significantly reduced the pain symptoms in TMJs and masseter muscles after six sessions of therapy.

However, the effect of the laser treatment (905 nm) was evaluated in two different therapeutic phases [24], being applied transversely to the TMJ for 240 s, and, respectively, to the lower and upper part of the masseter muscle, and anterior and posterior fascicles of the temporalis muscle, for a period of 60 s. The achieved therapeutic results indicated an improvement in pain intensity and frequency, associated with an increased capacity for mouth opening, after 12 laser treatment sessions. 

The effects of laser therapy were also compared to those achieved with needling, and the results showed no significant difference between the two therapeutic methods, with both being effective in treating the myofascial trigger points [20]. Other researchers studied the effect of low-level laser therapy (940 nm, 0.2 W) compared to occlusal splints for treating unilateral myogenic TMD. Their results showed that LLLT revealed a short-term therapeutic effect for treating unilateral myogenous TMD after 10 treatment sessions [23]. 

Therefore, all studies included in this review were randomized, controlled clinical trials published between 2013 and 2023 in English. Of the seven included studies, three studies [22,23,24] used gallium aluminum arsenide lasers (GaAlAs), one study used an infrared laser (Model Three Light) [20], one study used low-intensity semiconductor laser [19], and two studies did not mention the type of laser used. The shortest wavelength of the laser was 633 nm, and the longest wavelength was 940 nm. Power output ranged from 25 mW to 1500 mW. The laser was applied to the masseter muscles, temporalis muscles, and TMJ. All studies except one [24] used the VAS as an evaluation method for pain intensity. Two studies used sEMG signal analysis [20,23], two studies used a digital algometer for pressure pain threshold [19,24], and three studies evaluated mandibular movements [19,23,24]. This review found that five out of seven studies utilized placebo groups, one study [22] only used the experimental group, and another one compared the efficacy of LLLT with an occlusal splint [23].

To treat temporomandibular disorders, it is important to apply a validated diagnostic protocol, which involves exo- and endo-oral inspection and palpation of the muscles and TMJ, as well as measurements of mandibular movements and joint noise analysis. Another important aspect is the patient’s psychosocial status, which can be achieved using the Research Diagnostic Criteria for Temporomandibular Disorders (RDC/TMD), a diagnostic protocol that involves Axis I (physical assessment) and Axis II (biobehavioral questionnaire) [5]. Four studies utilized the RDC/TMD, one study used a numeric rating pain scale (NRPS) [24], and two studies used sEMG signal analysis, a bioelectrical signal produced during muscular activity [20,23,26].

The location of laser irradiation plays an important role in treating or managing pain in the temporomandibular region [15]. Targeting trigger points can alleviate the pain and induce overall comfort for the patient, compared to pre-established points. Several studies targeted the trigger and tender points according to the patient’s symptoms [18,19,20,22,23]. Others used three standard ipsilateral local points and one contralateral distal point at the TMJ level [21] or applied the laser in the first phase transversely throughout the temporomandibular joint region; in the second phase, a laser was applied to four predetermined points on the temporomandibular joint region [24].

The optimal laser range when it comes to reducing TMJ pain is between 830 and 904 nm [11], although two authors [18,22] used a combination of red and infrared wavelengths. Although none of the authors used the optimal wavelength range to alleviate TMJ pain, their findings showed an improvement in pain reduction when using laser wavelengths between 660 and 940 nm compared to placebo groups. These findings concluded that a specific wavelength alone could not improve TMJ symptoms, and further investigations need to be performed.

Each author used a different approach for laser frequency, ranging from a total of 3 sessions once a week [18], to 12 sessions 3 times per week [19], or 4 treatment sessions with different intervals between sessions [20], or even 1 treatment session per week until patients fully recovered [21], to 6 treatment sessions 3 times per week [22], 10 therapy sessions [23] or 12 sessions 3 times per week [24]. According to these findings, it is unclear whether frequent laser application could statistically improve pain symptoms compared to groups that received fewer laser applications. 

The ideal laser for treating temporomandibular joint (TMJ) pain should target specific trigger and tender points based on the patient’s symptoms rather than pre-established points to maximize pain relief and patient comfort. Several studies demonstrated the efficacy of this approach [18,19,20,22,23]. The optimal laser wavelength for reducing TMJ pain is within the range of 830 to 904 nm; however, none of the cited studies used this precise range. Despite this, wavelengths between 660 and 940 nm showed significant pain reduction compared to placebo groups. This suggests that while a specific wavelength might not be solely responsible for symptom improvement, the therapeutic range is broader and warrants further investigation. The frequency of laser application varied significantly across studies, from once a week for three weeks to twelve sessions over four weeks, indicating that there is no clear consensus on the optimal treatment frequency. Therefore, further research is needed to determine which type of laser is suitable when treating TMJ and muscular pain.

The study’s limitations are influenced by the lack of available articles, the divergence of opinions among them, and the absence of a standardized protocol. With a limited number of articles, the study’s findings are potentially less comprehensive, diminishing the overall accuracy of the conclusions. Divergent opinions further complicate the synthesis of findings, as conflicting viewpoints can obscure a clear consensus, making it challenging to draw definitive conclusions. Additionally, the lack of a standard protocol introduces variability in methodologies and results, reducing the ability to compare studies directly and systematically. The risk of bias assessment for five RCTs shows varied methodological rigor. Most studies have a low risk for random sequence generation but a high risk for allocation concealment. Performance and detection biases are generally low, although some studies show a high risk. All studies have a low risk for short-term attrition bias, with only one assessing long-term outcomes, also with a low risk. Selective reporting bias is low in most studies but high in a few. The quality assessment of Huang et al. [21] and Nencheva-Svechtarova et al. [22] shows both studies had clear objectives, large sample sizes, consistent interventions, and reliable outcome measures. However, uncertainties remain about participant representativeness and enrollment, with both studies lacking blinded outcome assessment and adequate follow-up accounting.

## 6. Conclusions

Although a clear protocol for the treatment of temporomandibular joint and muscular pain has not yet been established, the results of this study showed significant pain reduction with the use of low-level laser therapy. The effectiveness of LLLT can vary based on factors such as the type of laser used, treatment parameters (number of laser applications, total session time), and individual patient characteristics. Laser treatment is a non-invasive and harmless procedure that could be considered an alternative to other methods. Nevertheless, further investigation is necessary to establish a standardized procedure and compare its efficiency with other therapeutic alternatives such as physical therapy, medication, or even surgical procedures.

## Figures and Tables

**Table 1:** Combination of search terms used for each database.

Database	Keywords	Results
PubMed	(“laser s” [All Fields] OR “lasers” [MeSH Terms] OR “lasers” [All Fields] OR “laser” [All Fields] OR “lasered” [All Fields] OR “lasering” [All Fields]) AND (“therapeutics” [MeSH Terms] OR “therapeutics” [All Fields] OR “treatments” [All Fields] OR “therapy” [MeSH Subheading] OR “therapy” [All Fields] OR “treatment” [All Fields] OR “treatment s” [All Fields]) AND (“pain management” [MeSH Terms] OR (“pain” [All Fields] AND “management” [All Fields]) OR “pain management” [All Fields]) AND (((“TMD” [All Fields]) OR “temporomandibular joint disorders” [MeSH Terms] OR (“temporomandibular” [All Fields] AND “joint” [All Fields] AND “disorders” [All Fields]) OR “temporomandibular joint disorders” [All Fields] OR (“temporomandibular” [All Fields] AND “disorders” [All Fields]) OR “temporomandibular disorders” [All Fields] OR (“TMJ” [All Fields]) ) OR ( (“masticatory muscles”[MeSH Terms] OR (“masticatory” [All Fields] AND “muscles” [All Fields]) OR “masticatory muscles” [All Fields] OR (“masticatory” [All Fields] AND “muscle” [All Fields]) OR “masticatory muscle” [All Fields] OR “muscles” [All Fields]OR “masseter” [All Fields] OR “pterygoid” [All Fields] OR “temporal” [All Fields] OR “mandible” [All Fields] OR “maxilla” [All Fields]) AND (“pain” [MeSH Terms] OR “pain” [All Fields])))	93
Embase	(‘laser treatment’/exp OR ‘laser treatment’ OR ((‘laser’/exp OR laser) AND (‘treatment’/exp OR treatment))) AND (‘pain management’/exp OR ‘pain management’ OR ((‘pain’/exp OR pain) AND (‘management’/exp OR management))) AND (‘masticatory’ OR ‘masseter’ OR ‘pterygoid’ OR ‘temporomandibular joint disorders’ OR (temporomandibular AND (‘joint’/exp OR joint) AND (‘disorders’/exp OR disorders)))	124
Scopus	laser AND treatment AND pain AND management AND temporomandibular AND joint AND disorders AND masticatory AND muscle AND pain AND (LIMIT-TO (DOCTYPE, “ar”))	396
Web of Science	laser AND (treatment OR therapy OR management) AND (temporomandibular OR TMJ) AND (TMD OR masticatory OR masseter OR pterygoid OR temporal OR mandible OR maxilla OR muscle) AND pain	233

**Table 2:** Types of lasers and their characteristics used in different studies.

Author Year of Publication	Laser Type	Wavelength (nm)	Power Output (mW)	Time (Seconds)	Number of Laser Sessions/Number of Sessions Per Week/Number of Weeks
Al-Quisi et al. 2023 [18]	Red LED light	660 nm	1600 mW	300 s/point	3/1/4
	LASER	810 nm	1000 mW	30 s/point	3/1/4
Sancakli et al. 2015 [19]	Low-intensity semiconductor	820 nm	300 mW	10 s/point	12/3/4
Uemoto et al. 2013 [20]	Infrared laser (Model Three Light)	795 nm	80 mW	NM	4/2/2
Huang et al. 2014 [21]	Low-level energy diode laser	800 nm	1500 mW	134 s/point	NM/1/until recovery
Nencheva-Svechtarova et al. 2014 [22]	Gallium aluminum arsenide laser diodes	785 nm	3 × 33 mW	100 s/point	6/3/2
	SLD red light	633 nm	200 mW	300 s/point	6/3/2
Shousha et al. 2021 [23]	Gallium arsenide diode	940 nm	200 mW	10 s/point	10/3
Nambi et al. 2022 [24]	Gallium arsenide super pulsed laser	905 nm	25 mW	I: transversely throughout TMJ region for 240 s II: 60 s on 4 points on the TMJ region in the non-contact method	12/3/4

NM, not mentioned; SLD, superluminescent diode.

**Table 3:** Characteristics of the studies.

Authors Year of Publication	Study Type	Subjects	Evaluations	Evaluation Methods	Conclusions
Al-Quisi et al. 2023 [18]	randomized, and double-blind clinical trial	60	-week 0 and week 4	-VAS -RDC/TMD	-both red LED light and LASER therapy alleviated symptoms associated with TMDs -significant differences were found between their outcomes
Sancakli et al. 2015 [19]	randomized, controlled, double-blind clinical trial	30	-before the treatment and after the completion of the therapy	-VAS -mandibular movements -PPT -RDC/TMD	-a significant reduction in PPT (pain pressure threshold) values in both laser groups -improvement in mandibular movements in both laser groups -better results in the first laser group than in the second laser group
Uemoto et al. 2013 [20]	randomized controlled clinical trial	21	-after the 4th therapy session	-VAS -sEMG signal analysis -RDC/TMD	-best results were achieved using laser therapy combined with 2% lidocaine injection
Huang et al. 2014 [21]	controlled clinical trial	20	-each session	-VAS	-laser acupuncture was effective for the treatment of TMD
Nencheva-Svechtarova et al. 2014 [22]	clinical and experimental study	45	-before and after the completion of 6 sessions	-VAS -RDC/TMD	-significant reduction of pain symptoms
Shousha et al. 2021 [23]	double-blinded parallel group randomized controlled trial	112	-not mentioned	-temporomandibular joint opening index (TOI) -surface EMG (sEMG) -VAS	-short-term reduction of pain of the LLLT
Nambi et al. 2022 [24]	prospective, double-blinded, randomized	36	-at the end of 4th week, 8th week and 6th month follow up	-11 point numeric pain rating scale (NPRS) -pain threshold -mouth opening -temporomandibular joint disability index, -Quality of life (EuroQol 5D)	-ideal treatment protocol for TMDs combined with orofacial myalgia

**Table 4:** Cochrane Risk of Bias assessment for randomized controlled trials.

Criteria	Al-Quisi et al. 2023 [18]	Sancakli et al. 2015 [19]	Uemoto et al. 2013 [20]	Shousha et al. 2021 [23]	Nambi et al. 2022 [24]
Random sequence generation (selection bias)	low	low	high	low	low
Allocation concealment (selection bias)	high	high	high	low	high
Blinding of participants and personnel (performance bias)	low	low	high	low	high
Blinding of outcome assessment (detection bias) (patient-reported outcomes)	low	low	high	low	low
Blinding of outcome assessment (detection bias) (objective outcomes)	NA	low	high	low	low
Incomplete outcome data addressed (attrition bias) (Short-term outcomes)	low	low	low	low	low
Incomplete outcome data addressed (attrition bias) (Longer-term outcomes)	NA	NA	NA	NA	low
Selective reporting (reporting bias)	low	high	high	low	low

NA, not applicable.

**Table 5:** National Heart, Lung, and Blood Institute’s quality assessment tool for before-after studies with no control group.

Criteria	Huang et al. 2014 [21]	Nencheva-Svechtarova et al. 2014 [22]
1. Was the study question or objective clearly stated?	yes	yes
2. Were eligibility/selection criteria for the study population prespecified and clearly described?	yes	yes
3. Were the participants in the study representative of those who would be eligible for the test/service/intervention in the general or clinical population of interest?	CD	CD
4. Were all eligible participants that met the prespecified entry criteria enrolled?	CD	CD
5. Was the sample size sufficiently large to provide confidence in the findings?	yes	yes
6. Was the test/service/intervention clearly described and delivered consistently across the study population?	yes	yes
7. Were the outcome measures prespecified, clearly defined, valid, reliable, and assessed consistently across all study participants?	yes	yes
8. Were the people assessing the outcomes blinded to the participants’ exposures/interventions?	no	no
9. Was the loss to follow-up after baseline 20% or less? Were those lost to follow-up accounted for in the analysis?	yes	CD
10. Did the statistical methods examine changes in outcome measures from before to after the intervention? Were statistical tests done that provided *p* values for the pre-to-post changes?	yes	yes

CD, cannot determine.

## Data Availability

All research data is in the manuscript.

## References

[1] Wadhwa S., Kapila S. (2008). TMJ disorders: Future innovations in diagnostics and therapeutics. J. Dent. Educ..

[2] Okeson J.P., de Leeuw R. (2011). Differential diagnosis of temporomandibular disorders and other orofacial pain disorders. Dent. Clin. N. Am..

[3] Voß L.C., Basedau H., Svensson P., May A. (2024). Bruxism, temporomandibular disorders, and headache-a narrative review of correlations and causalities. Pain.

[4] Ostaș D., Almășan O., Ileșan R.R., Andrei V., Thieringer F.M., Hedeșiu M., Rotar H. (2022). Point-of-Care Virtual Surgical Planning and 3D Printing in Oral and Cranio-Maxillofacial Surgery: A Narrative Review. J. Clin. Med..

[5] Schiffman E., Ohrbach R., Truelove E., Look J., Anderson G., Goulet J.P., List T., Svensson P., Gonzalez Y., Lobbezoo F. (2014). International RDC/TMD Consortium Network, International Association for Dental Research; Orofacial Pain Special Interest Group, International Association for the Study of Pain. Diagnostic Criteria for Temporomandibular Disorders (DC/TMD) for Clinical and Research Applications: Recommendations of the International RDC/TMD Consortium Network* and Orofacial Pain Special Interest Group. J. Oral Fac. Pain Headache.

[6] Izzetti R., Carli E., Gennai S., Giuca M.R., Graziani F., Nisi M. (2024). Treatment Outcomes in Patients with Muscular Temporomandibular Joint Disorders: A Prospective Case-Control Study. Dent. J..

[7] Almășan O., Leucuța D.C., Buduru S. (2022). Disc Displacement of the Temporomandibular Joint and Facial Asymmetry in Children and Adolescents: A Systematic Review and Meta-Analysis. Children.

[8] Manziuc M.M., Almășan O., Kui A., Negucioiu M., Ispas A., Stupinean M., Varvară B., Buduru S. (2023). Temporomandibular disorders, occlusal splints, and treatment options: A survey-based investigation. Balneo PRM Res. J..

[9] Chan N.H.Y., Ip C.K., Li D.T.S., Leung Y.Y. (2022). Diagnosis and treatment of myogenous temporomandibular disorders: A clinical update. Diagnostics.

[10] Maia M.L., Bonjardim L.R., Quintans Jde S., Ribeiro M.A., Maia L.G., Conti P.C. (2012). Effect of low-level laser therapy on pain levels in patients with temporomandibular disorders: A systematic review. J. Appl. Oral Sci..

[11] Kumar L.S.V. (2014). Use of Lasers in the Management of Temporomandibular Disorders. Int. J. Laser Dent..

[12] Ahmad S.A., Hasan S., Saeed S., Khan A., Khan M. (2021). Low-level laser therapy in temporomandibular joint disorders: A systematic review. J. Med. Life.

[13] Zhang Y., Qian Y., Huo K., Liu J., Huang X., Bao J. (2023). Efficacy of laser therapy for temporomandibular disorders: A systematic review and meta-analysis. Complement Ther. Med..

[14] Wang X.D., Yang Z., Zhang W.H., Yi X.Z., Liang C.Y., Li X.J. (2011). Evaluation of the efficacy of low-intensity laser treatment for temporomandibular joint disorders. West China J. Stomatol..

[15] Shirani A.M., Gutknecht N., Taghizadeh M., Mir M. (2009). Low-level laser therapy and myofacial pain dysfunction syndrome: A randomized controlled clinical trial. Lasers Med. Sci..

[16] Alowaimer H.A., Al Shutwi S.S., Alsaegh M.K., Alruwaili O.M., Alrashed A.R., AlQahtani S.H., Batais M.S. (2024). Comparative Efficacy of Non-Invasive Therapies in Temporomandibular Joint Dysfunction: A Systematic Review. Cureus.

[17] Wu X., Zhu J., Zheng B., Liu J., Wu Z. (2021). Effectiveness of low-level gallium aluminium arsenide laser therapy for temporomandibular disorder with myofascial pain: A systemic review and meta-analysis. Medicine.

[18] Al-Quisi A.F., Jamil F.A., Abdulhadi B.N. (2023). The reliability of using light therapy compared with LASER in pain reduction of temporomandibular disorders: A randomized controlled trial. BMC Oral Health.

[19] Sancakli E., Gökçen-Röhlıg B., Balık A. (2015). Early results of low-level laser application for masticatory muscle pain: A double-blind randomized clinical study. BMC Oral Health.

[20] Uemoto L., Garcia M.A., Gouvêa C.V., Vilella O.V., Alfaya T.A. (2013). Laser therapy and needling in myofascial trigger point deactivation. J. Oral Sci..

[21] Huang Y.F., Lin J.C., Yang H.W., Lee Y.H., Yu C.H. (2014). Clinical effectiveness of laser acupuncture in the treatment of temporomandibular joint disorder. J. Formos. Med. Assoc..

[22] Nencheva-Svechtarova S., Svechtarov V., Gisbrecht A., Uzunov T.Z. (2014). Clinical and Experimental Study of Gaalas Phototherapy for Tемрoromandibular Disorders. Acta Med. Bulg..

[23] Shousha T., Alayat M., Moustafa I. (2021). Effects of low-level laser therapy versus soft occlusive splints on mouth opening and surface electromyography in females with temporomandibular dysfunction: A randomized-controlled study. PLoS ONE..

[24] Nambi G., Abdelbasset W.K., Soliman G.S., Alessi A.A., Alsalem I.N., Ali Z.A. (2022). Clinical and functional efficacy of gallium–arsenide super pulsed laser therapy on temporo mandibular joint pain with orofacial myalgia following healed unilateral cervicofacial burn—A randomized trial. Burns.

[25] Badel T., Zadravec D., Bašić Kes V., Smoljan M., Kocijan Lovko S., Zavoreo I., Krapac L., Anić Milošević S. (2019). Orofacial pain-diagnostic and therapeutic challenges. Acta Clin. Croat..

[26] Wu J., Li X., Liu W., Wang Z.J. (2019). sEMG signal processing methods: A review. J. Phys. Conf. Ser..

